# Supplementary material for: Deep learning analysis of epicardial adipose tissue to predict cardiovascular risk in heavy smokers
Source: Commun Med (Lond). 2024 Mar 13;4:44. doi: 10.1038/s43856-024-00475-1 (PMC10937640; doi:10.1038/s43856-024-00475-1)
Supplement: Supplementary file 2 — Supplementary information [file 43856_2024_475_MOESM2_ESM.pdf]

Deep Learning Analysis of Epicardial Adipose Tissue to Predict Cardiovascular Risk in Heavy Smokers

Running title: Epicardial fat & cardiovascular risk on non-gated chest CT

Borek Foldyna[1], Ibrahim Hadzic[2,3,4], Roman Zeleznik[2,3], Marcel C Langenbach[1,5], Vineet K Raghu[1], Thomas Mayrhofer[1,6], Michael T Lu[1], Hugo JWL Aerts[2,3,4]

[1] Cardiovascular Imaging Research Center (CIRC), Massachusetts General Hospital – Harvard Medical School, Boston, USA

[2] Artificial Intelligence in Medicine (AIM) Program, Mass General Brigham, Harvard Medical School, Boston, Massachusetts, USA

[3] Department of Radiation Oncology, Brigham and Women's Hospital, Dana-Farber Cancer Institute, Harvard Medical School, Boston, USA

[4] Radiology and Nuclear Medicine, CARIM & GROW, Maastricht University, Maastricht, The Netherlands

[5] Institute for Diagnostic and Interventional Radiology, University Hospital Cologne, Cologne, Germany

[6] School of Business Studies, Stralsund University of Applied Sciences, Stralsund, Germany

Supplementary Information

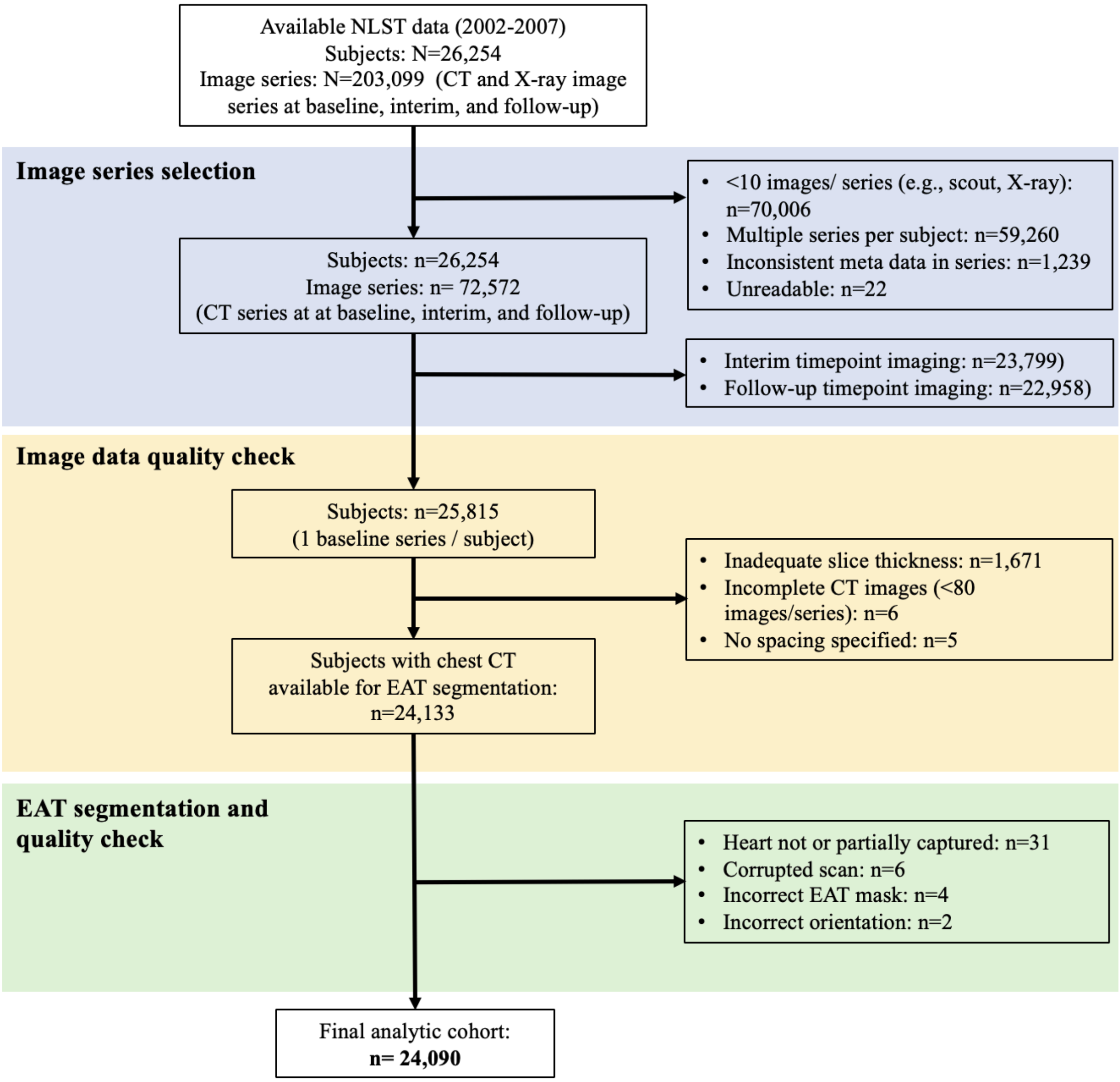

Supplementary Figure 1. Participant flow diagram. CT = computed tomography, EAT = epicardial adipose tissue, NLST = National Lung Screening Trial.

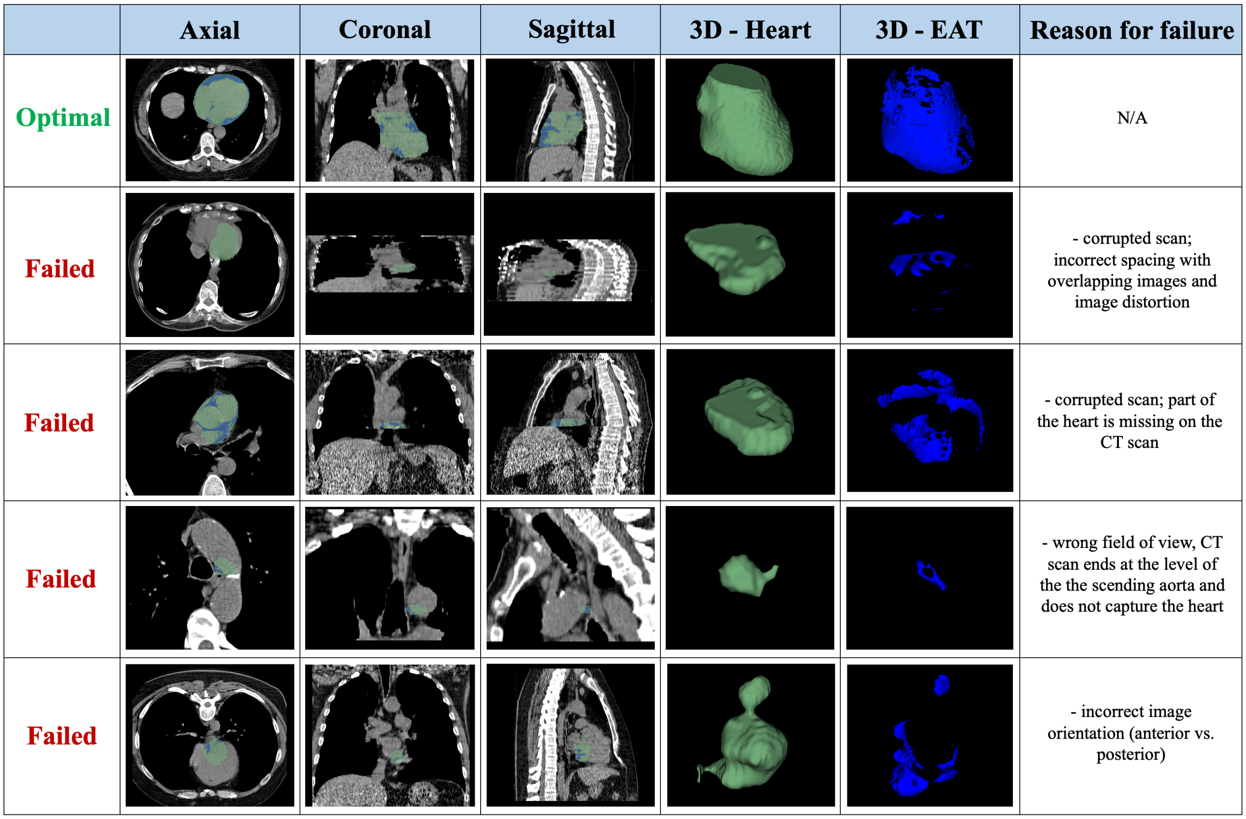

Supplementary Figure 2. Examples of successful and failed EAT segmentations. CT= computed tomography; EAT = epicardial adipose tissue. CT = computed tomography, EAT = epicardial adipose tissue.

Supplementary Table 1. Event distribution across quartiles of EAT volume and density

		All-cause death			CV death		
	**EAT volume –cm^3/m^2**	**No**	**Yes**	***P***	**No**	**Yes**	***P***
**Q1**	42.2±8.1	5,024 (83.7)	978 (16.3)	<0.001	5,782 (96.3)	220 (3.7)	<0.001
**Q2**	60.2±4.2	5,042 (84.0)	960 (16.0)		5,803 (96.7)	199 (3.3)	
**Q3**	75.5±5.0	4,824 (80.4)	1,178 (19.6)		5,719 (95.3)	283 (4.7)	
**Q4**	103.4±17.2	4,450 (74.1)	1,552 (25.9)		5,621 (93.7)	381 (6.4)	
	**EAT density –HU**						
**Q1**	-84.3±2.6	4,697 (78.0)	1,326 (22.0)	<0.001	5,711 (94.8)	312 (5.2)	0.047
**Q2**	-79.3±1.0	4,862 (80.7)	1,160 (19.3)		5,760 (95.7)	262 (4.4)	
**Q3**	-75.9±1.0	4,940 (82.0)	1,083 (18.0)		5,768 (95.8)	255 (4.2)	
**Q4**	-71.1±2.3	4,901 (81.4)	1,121 (18.6)		5,762 (95.7)	260 (4.3)	

CV = cardiovascular, EAT = epicardial adipose tissue.

Supplementary Table 2. Association of EAT measures with all-cause and cardiovascular mortality in a subgroup of participants **without known CV disease** (coronary artery disease, heart failure, or stroke**)** at baseline (n=20,454; all-cause mortality: n=3,539; CV death: n=719).

	**All-cause mortality**									**Cardiovascular mortality**								
	**Model 1 (bivariable)**			**Model 2**			**Model 3**			**Model 1 (bivariable)**			**Model 2**			**Model 3**		
	**HR**	**95%CI**	***P***	**HR**	**95%CI**	***P***	**HR**	**95%CI**	***P***	**HR**	**95%CI**	***P***	**HR**	**95%CI**	***P***	**HR**	**95%CI**	***P***
EAT volume –per 10 cm^3/m^2	1.19	1.17–1.21	<0.001	1.11	1.09–1.13	<0.001	1.11	1.08–1.14	<0.001	1.25	1.21–1.31	<0.001	1.15	1.10–1.20	<0.001	1.14	1.08–1.20	<0.001
EAT density –per 10 HU	1.71	1.55–1.88	<0.001	1.37	1.24–1.51	<0.001	1.37	1.21–1.55	<0.001	2.16	1.76–2.65	<0.001	1.75	1.42–2.15	<0.001	1.81	1.38–2.38	<0.001
Age –years				1.09	1.08–1.10	<0.001	1.08	1.07–1.09	<0.001				1.10	1.09–1.12	<0.001	1.08	1.06–1.10	<0.001
Male sex				1.29	1.20–1.39	<0.001	1.22	1.11–1.34	<0.001				1.34	1.14–1.57	<0.001	1.14	0.92–1.42	0.235
Race																		
White				Ref.	-	-	Ref.	-	-				Ref.	-	-	Ref.	-	-
African American				1.40	1.20–1.63	<0.001	1.42	1.17–1.75	0.001				1.66	1.22–2.25	0.001	1.78	1.17–2.73	0.008
Asian				0.65	0.51–0.85	0.001	0.62	0.44–0.86	0.004				0.75	0.43–1.30	0.299	0.69	0.32–1.46	0.328
Ethnicity																		
Hispanic/Latinx				Ref.	-	-	Ref.	-	-				Ref.	-	-	Ref.	-	-
Non-Hispanic/Latinx				1.38	1.04–1.84	0.025	1.31	0.91–1.87	0.142				1.19	0.67–2.11	0.545	1.28	0.59–2.77	0.540
Smoking																		
Former				Ref.	-	-	Ref.	-	-				Ref.	-	-	Ref.	-	-
Current				1.89	1.76–2.0	<0.001	1.82	1.66–2.0	<0.001				2.00	1.72–2.34	<0.001	1.89	1.54–2.33	<0.001
Packyears (per 1 pack year)				1.01	1.01–1.01	<0.001	1.01	1.01–1.01	<0.001				1.01	1.00–1.01	<0.001	1.01	1.00–1.01	0.001
Diabetes				1.59	1.44–1.77	<0.001	1.51	1.31–1.73	<0.001				1.92	1.56–2.36	<0.001	1.58	1.19–2.09	0.002
Hypertension				1.19	1.11–1.28	<0.001	1.11	1.01–1.22	0.029				1.46	1.25–1.71	<0.001	1.30	1.06–1.60	0.013
Education																		
High school graduate or below				Ref.	-	-	Ref.	-	-				Ref.	-	-	Ref.	-	-
Post high school (excl. college)				0.91	0.82–1.01	0.071	0.93	0.82–1.07	0.316				0.81	0.63–1.04	0.094	0.98	0.72–1.33	0.889
Some college or bachelor's degree				0.79	0.73–0.85	<0.001	0.77	0.70–0.86	<0.001				0.88	0.74–1.05	0.164	0.86	0.68–1.08	0.196
Graduate school				0.69	0.62–0.77	<0.001	0.65	0.56–0.76	<0.001				0.86	0.67–1.09	0.201	0.70	0.50–0.99	0.042
BMI (per 1 kg/m^2)				0.98	0.97–0.99	<0.001	0.98	0.97–0.99	<0.001				1.01	0.99–1.02	0.513	1.02	1.00–1.05	0.021

*CAC score was available in a subgroup of 13,966 participants. BMI = body mass index, CAC = coronary artery calcium, EAT = epicardial adipose tissue, MI = myocardial infarction.

Supplementary Table 3. Association of EAT measures with all-cause and cardiovascular mortality in a subgroup of participants **without CAC** at baseline (n=3,355)

	**All-cause mortality**						**Cardiovascular mortality**					
	**Model 1 (bivariable)**			**Model 2**			**Model 1 (bivariable)**			**Model 2**		
	**HR**	**95%CI**	***P***	**HR**	**95%CI**	***P***	**HR**	**95%CI**	***P***	**HR**	**95%CI**	***P***
EAT volume –per 10 cm^3/m^2	1.19	1.12–1.26	<0.001	1.10	1.03–1.17	0.003	1.33	1.17–1.51	<0.001	1.21	1.05–1.40	0.01
EAT density –per 10 HU	1.33	1.00–1.77	0.05	1.11	0.83–1.49	0.488	2.05	1.03–4.09	0.042	1.83	0.90–3.71	0.09
Age –years				1.09	1.06–1.11	<0.001				1.10	1.05–1.16	<0.001
Male sex				1.45	1.17–1.79	0.001				1.08	0.64–1.83	0.77
Race												
White				Ref.	-	-				Ref.	-	-
African American				1.53	1.03–2.27	0.03				3.58	1.64–7.82	0.001
Asian				0.59	0.28–1.27	0.18				0.74	0.10–5.63	0.774
Ethnicity												
Hispanic/Latinx				Ref.	-	-				Ref.	-	-
Non-Hispanic/Latinx				0.83	0.40–1.72	0.611				0.64	0.14–2.96	0.567
Smoking												
Former				Ref.	-	-				Ref.	-	-
Current				1.66	1.34–2.05	<0.001				1.83	1.08–3.12	0.03
Packyears (per 1 pack year)				1.01	1.00–1.01	<0.001				1.01	1.00–1.02	0.04
History of heart disease				1.68	1.20–2.37	<0.001				4.19	2.18–8.04	<0.001
History of stroke				1.05	0.58–1.89	0.882				0.89	0.21–3.87	0.878
Diabetes				1.76	1.28–2.42	0.001				1.70	0.82–3.53	0.153
Hypertension				1.03	0.82–1.29	0.807				0.67	0.38–1.20	0.178
Education												
High school graduate or below				Ref.	-	-				Ref.	-	-
Post high school (excl. college)				1.08	0.80–1.47	0.646				1.22	0.55–2.72	0.630
Some college or bachelor's degree				0.85	0.67–1.08	0.184				1.13	0.62–2.06	0.680
Graduate school				0.61	0.42–0.89	0.01				0.53	0.18–1.58	0.258
BMI (per 1 kg/m^2)				0.98	0.98–1.00	0.09				1.03	0.98–1.09	0.198

BMI = body mass index, EAT = epicardial adipose tissue.

Supplementary Table 4. Association of EAT volume and density with all-cause and cardiovascular mortality

	All-cause mortality									Cardiovascular mortality								
	Model 1 (bivariable)			Model 2			Model 3			Model 1 (bivariable)			Model 2			Model 3		
	HR	95%CI	*P*	HR	95%CI	*P*	HR	95%CI	*P*	HR	95%CI	*P*	HR	95%CI	*P*	HR	95%CI	*P*
EAT volume –per 10 cm^3/m^2	1.19	1.17–1.21	<0.001	1.10	1.08–1.12	<0.001	1.10	1.08–1.13	<0.001	1.27	1.23–1.30	<0.001	1.15	1.11–1.19	<0.001	1.14	1.10–1.19	<0.001
EAT density –per 10 HU	1.66	1.53–1.80	<0.001	1.36	1.26–1.48	<0.001	1.38	1.24–1.54	<0.001	2.14	1.81–2.52	<0.001	1.76	1.49–2.09	<0.001	1.78	1.42–2.22	<0.001
Age –years				1.09	1.08–1.10	<0.001	1.08	1.07–1.09	<0.001				1.09	1.08–1.11	<0.001	1.08	1.06–1.10	<0.001
Male sex				1.28	1.20–1.37	<0.001	1.22	1.12–1.32	<0.001				1.39	1.21–1.59	<0.001	1.17	0.97–1.40	0.10
Race																		
White				Ref.	-	-	Ref.	-	-				Ref.	-	-	Ref.	-	-
African American				1.35	1.18–1.54	<0.001	1.39	1.17–1.66	<0.001				1.69	1.33–2.16	<0.001	1.94	1.41–2.68	<0.001
Asian				0.62	0.50–0.78	<0.001	0.63	0.47–0.84	0.002				0.68	0.42–1.08	0.10	0.74	0.40–1.35	0.33
Ethnicity																		
Hispanic/Latinx				Ref.	-	-	Ref.	-	-				Ref.	-	-	Ref.	-	-
Non-Hispanic/Latinx				1.51	1.17–1.96	0.002	1.46	1.06–2.02	0.02				1.32	0.81–2.17	0.26	1.62	0.82–3.19	0.16
Smoking																		
Former				Ref.	-	-	Ref.	-	-				Ref.	-	-	Ref.	-	-
Current				1.82	1.71–1.93	<0.001	1.78	1.64–1.92	<0.001				1.95	1.72–2.21	<0.001	2.03	1.72–2.40	<0.001
Pack years (per 1 pack year)				1.01	1.01–1.01	<0.001	1.01	1.01–1.01	<0.001				1.01	1.00–1.01	<0.001	1.01	1.00–1.01	0.001
History of heart disease				1.46	1.35–1.57	<0.001	1.31	1.19–1.45	<0.001				2.01	1.75–2.31	<0.001	1.72	1.42–2.07	<0.001
History of stroke				1.44	1.26–1.65	<0.001	1.41	1.19–1.67	<0.001				2.08	1.66–2.60	<0.001	1.88	1.40–2.53	<0.001
Diabetes				1.63	1.50–1.77	<0.001	1.53	1.37–1.71	<0.001				1.78	1.52–2.08	<0.001	1.51	1.22–1.87	<0.001
Hypertension				1.19	1.12–1.26	<0.001	1.11	1.03–1.21	0.008				1.39	1.23–1.58	<0.001	1.26	1.06–1.49	0.007
Education																		
High school graduate or below				Ref.	-	-	Ref.	-	-				Ref.	-	-	Ref.	-	-
Post high school (excl. college)				0.93	0.85–1.02	0.105	0.94	0.84–1.06	0.29				0.87	0.72–1.05	0.15	0.99	0.78–1.26	0.95
Some college or bachelor's degree				0.80	0.75–0.86	<0.001	0.77	0.70–0.84	<0.001				0.85	0.74–0.99	0.03	0.80	0.66–0.97	0.02
Graduate school				0.71	0.64–0.78	<0.001	0.68	0.60–0.77	<0.001				0.78	0.64–0.99	0.02	0.67	0.51–0.88	0.004
BMI (per 1 kg/m^2)				0.98	0.97–0.99	<0.001	0.99	0.98–1.00	0.001				1.01	1.00–1.02	0.16	1.03	1.01–1.04	0.002
CAC score																		
0							Ref.	-	-							Ref.	-	-
1–100							1.22	1.08–1.38	0.002							1.26	0.92–1.71	0.15
101–300							1.41	1.23–1.62	<0.001							1.84	1.33–2.54	<0.001
>300							1.71	1.51–1.94	<0.001							2.78	2.08–3.70	<0.001

BMI = body mass index, CAC = coronary artery calcium, EAT = epicardial adipose tissue, MI = myocardial infarction.
