# Supplementary material for: Deep learning analysis of epicardial adipose tissue to predict cardiovascular risk in heavy smokers
Source: Commun Med (Lond). 2024 Mar 13;4:44. doi: 10.1038/s43856-024-00475-1 (PMC10937640; doi:10.1038/s43856-024-00475-1)
Supplement: Supplementary file 3 — Description of Additional Supplementary Files [file 43856_2024_475_MOESM3_ESM.pdf]

Description of Additional Supplementary Files

File Name: Supplementary Data 1
Description: Table: EAT volume and density across clinical characteristics

File Name: Supplementary Data 2
Description: Source data (EAT volume and density measurements)
