# Supplementary material for: Deep learning analysis of epicardial adipose tissue to predict cardiovascular risk in heavy smokers
Source: Commun Med (Lond). 2024 Mar 13;4:44. doi: 10.1038/s43856-024-00475-1 (PMC10937640; doi:10.1038/s43856-024-00475-1)
Supplement: Supplementary file 6 — Reporting summary [file 43856_2024_475_MOESM6_ESM.pdf]

nature portfolio

Corresponding author(s): Borek Foldyna

Last updated by author(s): Feb 29, 2024

Reporting Summary

Nature Portfolio wishes to improve the reproducibility of the work that we publish. This form provides structure for consistency and transparency in reporting. For further information on Nature Portfolio policies, see our Editorial Policies and the Editorial Policy Checklist.

Statistics

For all statistical analyses, confirm that the following items are present in the figure legend, table legend, main text, or Methods section.

n/a	Confirmed	
☐	☒	The exact sample size (*n*) for each experimental group/condition, given as a discrete number and unit of measurement
☐	☒	A statement on whether measurements were taken from distinct samples or whether the same sample was measured repeatedly
☐	☒	The statistical test(s) used AND whether they are one- or two-sided<br>*Only common tests should be described solely by name; describe more complex techniques in the Methods section.*
☐	☒	A description of all covariates tested
☐	☒	A description of any assumptions or corrections, such as tests of normality and adjustment for multiple comparisons
☐	☒	A full description of the statistical parameters including central tendency (e.g. means) or other basic estimates (e.g. regression coefficient) AND variation (e.g. standard deviation) or associated estimates of uncertainty (e.g. confidence intervals)
☐	☒	For null hypothesis testing, the test statistic (e.g. *F*, *t*, *r*) with confidence intervals, effect sizes, degrees of freedom and *P* value noted<br>*Give P values as exact values whenever suitable.*
☒	☐	For Bayesian analysis, information on the choice of priors and Markov chain Monte Carlo settings
☐	☒	For hierarchical and complex designs, identification of the appropriate level for tests and full reporting of outcomes
☐	☒	Estimates of effect sizes (e.g. Cohen's *d*, Pearson's *r*), indicating how they were calculated

Our web collection on statistics for biologists contains articles on many of the points above.

Software and code

Policy information about availability of computer code

Data collection	NLST data, including raw CT images, are available per request from the National Cancer Institute at https://biometry.nci.nih.gov/cdas/nlst.
Data analysis	STATA SE 17.0 for statistical analysis, Development and evaluation were done on a Linux workstation equipped with one Nvidia RTX A6000 GPU using Tensorflow-GPU (v1.14), Keras (v2.3.1), and NVIDIA CUDA (v10.2).

For manuscripts utilizing custom algorithms or software that are central to the research but not yet described in published literature, software must be made available to editors and reviewers. We strongly encourage code deposition in a community repository (e.g. GitHub). See the Nature Portfolio guidelines for submitting code & software for further information.

Data

Policy information about availability of data

All manuscripts must include a data availability statement. This statement should provide the following information, where applicable:

- Accession codes, unique identifiers, or web links for publicly available datasets
- A description of any restrictions on data availability
- For clinical datasets or third party data, please ensure that the statement adheres to our policy

NLST data, including raw CT images, are available per request from the National Cancer Institute at https://biometry.nci.nih.gov/cdas/nlst. Although raw CT imaging data cannot be shared, the derived EAT volume and density are shared as Supplementary Data 2.

Human research participants

Policy information about studies involving human research participants and Sex and Gender in Research.

Reporting on sex and gender	Sex variable was used for all analyses as coded in the main NLST trial data set.
Population characteristics	Adult heavy smokers enrolled in the NLST trial.
Recruitment	Secondary analysis of the NLST trial.
Ethics oversight	The secondary use of NLST data was approved by the National Cancer Institute (Bethesda, Maryland) and the local Mass General Brigham (Boston, MA) institutional review board. All participants provided written consent to the parent trial, and informed consent was waived for this retrospective study.

Note that full information on the approval of the study protocol must also be provided in the manuscript.

Field-specific reporting

Please select the one below that is the best fit for your research. If you are not sure, read the appropriate sections before making your selection.

☒ Life sciences ☐ Behavioural & social sciences ☐ Ecological, evolutionary & environmental sciences

For a reference copy of the document with all sections, see nature.com/documents/nr-reporting-summary-flat.pdf

Life sciences study design

All studies must disclose on these points even when the disclosure is negative.

Sample size	Secondary analysis of all available NLST participants.
Data exclusions	Incomplete or corrupt image data. Incomplete clinical data
Replication	N/A
Randomization	Secondary analysis of the NLST trial.
Blinding	N/A

Reporting for specific materials, systems and methods

We require information from authors about some types of materials, experimental systems and methods used in many studies. Here, indicate whether each material, system or method listed is relevant to your study. If you are not sure if a list item applies to your research, read the appropriate section before selecting a response.

Materials & experimental systems

n/a	Involved in the study	
☒	☐	Antibodies
☒	☐	Eukaryotic cell lines
☒	☐	Palaeontology and archaeology
☒	☐	Animals and other organisms
☐	☒	Clinical data
☒	☐	Dual use research of concern

Methods

n/a	Involved in the study	
☒	☐	ChIP-seq
☒	☐	Flow cytometry
☒	☐	MRI-based neuroimaging

Clinical data

Policy information about clinical studies

All manuscripts should comply with the ICMJE guidelines for publication of clinical research and a completed CONSORT checklist must be included with all submissions.

Clinical trial registration	NCT00047385

Study protocol	N/A
Data collection	NLST data, including raw CT images, are available per request from the National Cancer Institute at https://biometry.nci.nih.gov/cdas/nlst. Although raw CT imaging data cannot be shared, all measured results to replicate the statistical analysis in NLST are shared on the Artificial Intelligence in Medicine (AIM) webpage at aim.hms.harvard.edu. Furthermore, we include test samples from a publicly available data set with deep learning and expert reader epicardial adipose tissue segmentations.
Outcomes	Included in the NLST data set.
